# A commentary on ‘Management of leaks following one-anastomosis gastric bypass: an updated systematic review and meta-analysis of 44 318 patients’

**DOI:** 10.1097/JS9.0000000000000635

**Published:** 2023-08-04

**Authors:** Chao-Ming Hung, Chi-Ming Tai, Chong-Chi Chiu

**Affiliations:** a Department of General Surgery; b Division of Gastroenterology and Hepatology, Department of Internal Medicine; c Department of Medical Education and Research, E-Da Cancer Hospital; d School of Medicine for International Students; e School of Medicine, College of Medicine, I-Shou University, Kaohsiung, Taiwan

*Dear Editor*,

The limited occurrence of leakage following laparoscopic one-anastomosis gastric bypass (OAGB) may explain the scarcity of literature on the presentation and treatment of this lethal complication in contrast to other bariatric procedures. Given the lack of substantial expertise in managing anastomotic leaks in bariatric patients, the perspectives shared by Kermansaravi *et al*.^1^ should raise concerns among bariatric surgeons.

Leakage remains the leading cause of 30-day mortality, accounting for 48% of all early postoperative deaths in bariatric surgeries. However, the clinical presentation of gastrointestinal leaks may be less apparent or delayed in obese patients compared to individuals of average weight, making timely diagnosis challenging. Current knowledge suggests clinicians cannot always predict this complication. Detecting and treating this postoperative complication in high-risk patients is crucial, and early detection improves both quality outcomes and patient prognosis.

In clinical practice, surgeons rely mainly on early clinical evolution and serum biomarkers to make decisions regarding the early discharge of bariatric patients. However, we should alert the possibility of leakage if a bariatric patient presents with constant abdominal pain, tachycardia (>120 beats per minute), dyspnea, and even fever. Ordinarily, the clinical signs related to anastomosis leakage manifest generally 3 days following the surgery. Additionally, Shi *et al*.^2^ pointed out that C-reactive protein (CRP) is a reliable parameter for the earlier discovery of the leakage emergency for bariatric patients, independent of their clinical signs. In bariatric patients with a CRP value lower than 70 mg/l on the first postoperative day, surgeons could accurately exclude early intra-abdominal infection. In other words, clinicians could use early postoperative CRP levels to estimate the risk of anastomotic leakage within the Enhanced Recovery After Surgery (ERAS) program. Due to a high negative predictive efficacy, clinicians could discharge the patients on the fifth postoperative day if the CRP levels were lower than the cut-off value. On the contrary, we recommend that those patients with a high early postoperative CRP level receive nothing by mouth and undergo detailed imaging studies immediately before clinical deterioration.

Taking into account the individual clinical situations (presentation of stability or sepsis) and results from oral contrast computed tomography (CT) scans (evidence of leakage, involved area, and abscess location), we agree with the recommendation by Kermansaravi *et al*.^1^ to develop a leak management algorithm. Kalff *et al*.^3^ even emphasized that CT scans can reduce re-operation rates in stable patients. While most OAGB cases involve leakage via the gastrojejunal anastomosis (GJA) or gastric tube (GT), leakage from other possible areas, including the residual stomach, efferent or afferent limbs, or colon, has been addressed. Although Roux-en-Y gastric bypass (RYGB) shows promising results for acute leaks after OAGB, with better outcomes and shorter hospital stays, nearly 54% of OAGB surgeons reported rarely or never performing RYGB^4^.

Liagre *et al*.^5^ highlighted that lower pressure in the GT part and bile content within the afferent limb are two primary factors in determining the appropriate strategy, as the small intestine provides a preferred pathway for the leak to seep into. In stable patients, we recommend percutaneous abscess drainage as the initial approach, as it is often sufficient to achieve spontaneous closure of leaks, particularly in cases with low pressure in the GT part. Additionally, if the leakage point is located at the anterior side of the GJA and is sufficiently large, we prefer a direct percutaneous T-tube insertion via the leaking GJA orifice. Moreover, the leakage may manifest as a tiny orifice with a low output in some patients. In our experience, we noticed some leaking orifices had healed already during the surgical re-exploration. In such situations, if the interventional radiologist and endoscopist are accessible, re-operation can be averted in many clinically stable patients. However, continuous patient monitoring and the ability to provide immediate management are essential when we choose a minimally invasive strategy instead. Finally, immediate conversion to RYGB is reserved for the condition with leaking points at the posterior side of the GJA due to the inaccessibility of intubation. Although some bariatric surgeons have advocated the application of biologic tissue sealants for GJA leaks, further research may help clarify their effectiveness.

Managing leaks requires a multidisciplinary technical platform, necessitating collaboration among the emergency room, intensive care unit, gastrointestinal endoscopy, interventional radiology, and an expert bariatric surgical team. However, many OAGB surgeons must become familiar with the conversion technique of OAGB to RYGB.

## Ethical approval

This is only a commentary, not research involving patients. No ethical approval is required.

## Consent

This is only a commentary, not research involving patients. No patient consent is required.

## Sources of funding

This is a commentary. We have no funding for our commentary.

## Author contribution

C.-M.H.: conceptualization; C.-M.T.: validation; C.-C.C.: conceptualization, writing original draft, supervision, submission, and correspondence.

## Conflicts of interest disclosure

The authors declare that they have no conflicts of interest.

## Research registration unique identifying number (UIN)

This is a commentary on a study published in ‘International Journal of Surgery’. No UIN is required.

## Guarantor

Professor Chong-Chi Chiu.

## Data availability statement

This is only a commentary on a study published in ‘International Journal of Surgery’. There is no research data in our commentary.

## Provenance and peer review

This paper was not invited.

## References

[1] Kermansaravi M Kassir R Valizadeh R. Management of leaks following one-anastomosis gastric bypass: an updated systematic review and meta-analysis of 44 318 patients. Int J Surg 2023;109:1497–508. 37026835 10.1097/JS9.0000000000000346 PMC10389517

[2] Shi J Wu Z Wang Q. Clinical predictive efficacy of C-reactive protein for diagnosing infectious complications after gastric surgery. Therap Adv Gastroenterol 2020;13:1756284820936542. 10.1177/1756284820936542 PMC7339084 32670413

[3] Kalff MC de Raaff CAL de Vries CEE. Diagnostic value of computed tomography for detecting anastomotic or staple line leakage after bariatric surgery. Surg Obes Relat Dis 2018;14:1310–1316. 30580770 10.1016/j.soard.2018.05.007

[4] Haddad A Bashir A Fobi M. The IFSO worldwide one anastomosis gastric bypass survey: techniques and outcomes? Obes Surg 2021;31:1411–21. 33517557 10.1007/s11695-021-05249-5

[5] Liagre A Queralto M Juglard G. Multidisciplinary management of leaks after one-anastomosis gastric bypass in a single-center series of 2780 consecutive patients. Obes Surg 2019;29:1452–61. 30726544 10.1007/s11695-019-03754-2

